# Supplementary material for: Induction of resident memory T cells enhances the efficacy of cancer vaccine
Source: Nat Commun. 2017 May 24;8:15221. doi: 10.1038/ncomms15221 (PMC5458068; doi:10.1038/ncomms15221)
Supplement: Supplementary Information — Supplementary Figures and Supplementary Tables [file ncomms15221-s1.pdf]

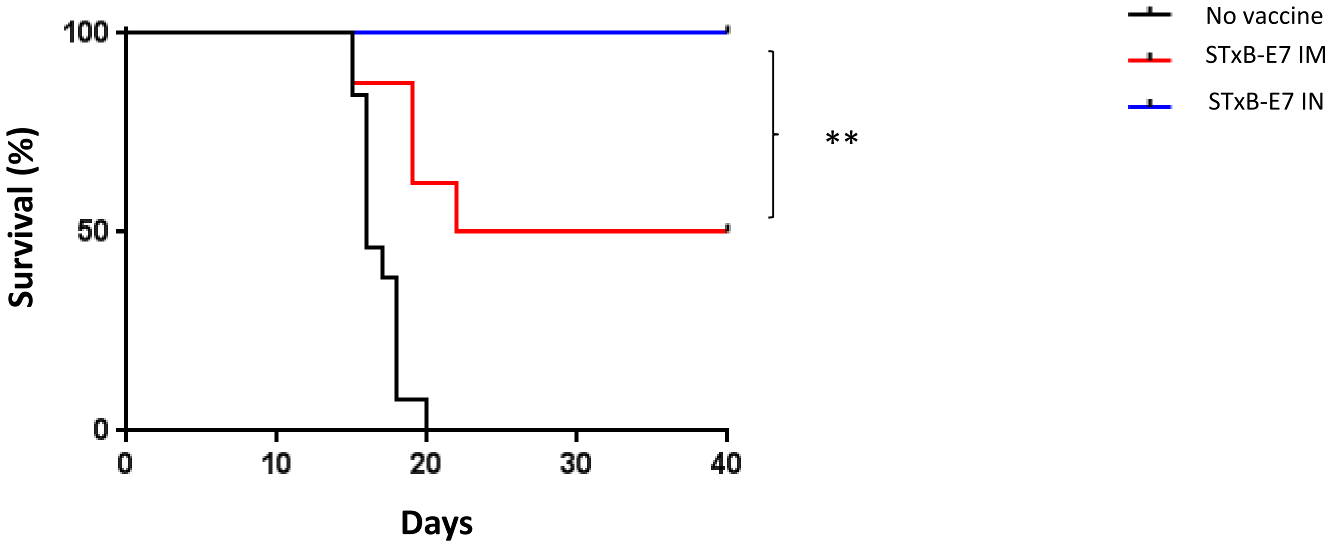

Supplementary Figure 1 : Comparative analysis of the route of immunization on the efficacy of cancer vaccine on an orthotopic head and neck tumor.

Mice (n = 5) were grafted in the tongue with the TC1 cells (D0) and then administered via the i.n or the i.m route with two doses of the STxB-E7 vaccine + α-GalCer at D9 and D14. Kaplan-Meier survival curve of i.n. (STxB-E7 IN) versus i.m. (STxB-E7 IM) and non immunized mice. Experiment were reproduced three times. Log-rank test was selected for survival analysis **p<0,01

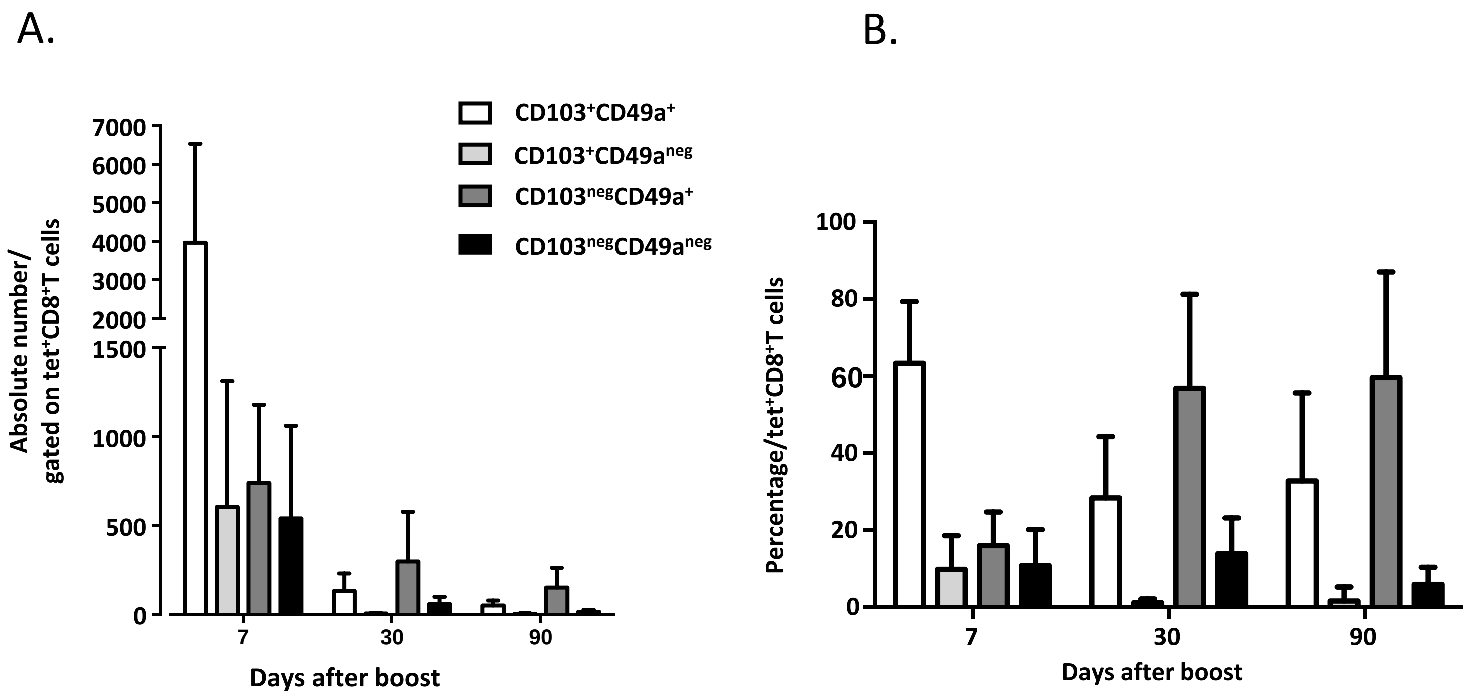

Supplementary Figure 2 : Kinetics of Trm cell subpopulations after i.n immunization

Mice were i.n. immunized (prime-boost) with STxB-E7 and BAL were collected at D7, D30 and D90 after the boost. Cells were labeled with anti-CD8, anti-CD103, anti-CD49a and D^b $E7_{39\text{-}47}$ tetramer. Absolute numbers (A) and percentages (B) of the various subpopulations of anti-$E7_{39\text{-}47}$ Trm after gating on D^b-$E7_{39\text{-}47}$-tetramer$^+$ $CD8^+$Tcells and of non Trm cells ($CD103^{neg}CD49a^{neg}$). Values are expressed as means + SEM. The results are representative of two experiments (n=4/experiment).

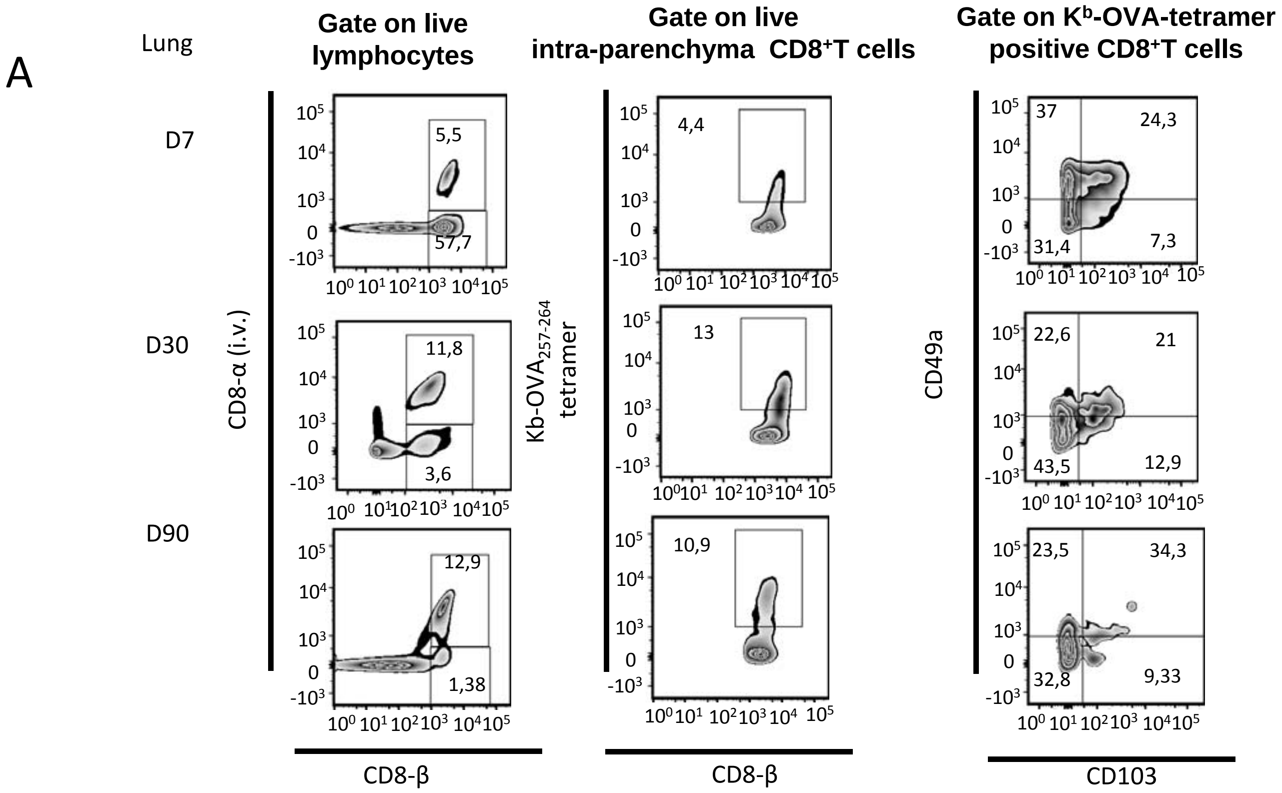

A
Lung
D7
D30
D90
Gate on live lymphocytes
Gate on live intra-parenchyma CD8+T cells
Gate on Kb-OVA-tetramer positive CD8+T cells
CD8-α (i.v.)
Kb-OVA257-264 tetramer
CD49a
CD8-β
CD8-β
CD103
5,5
57,7
11,8
3,6
12,9
1,38
4,4
13
10,9
37
24,3
31,4
7,3
22,6
21
43,5
12,9
23,5
34,3
32,8
9,33

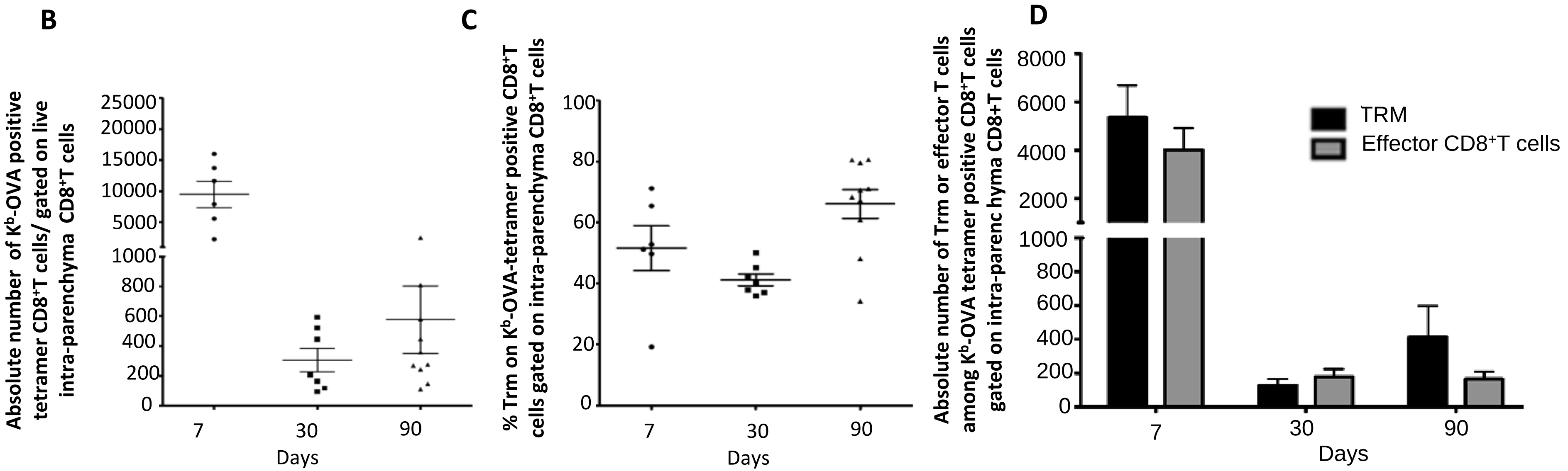

Supplementary Figure 3: Persistence of resident memory CD8$^+$T cells in the lung after intranasal vaccine administration

Mice (n = 4) were i.n. immunized (prime (D0) – boost (D14)) with STxB-OVA and the lung were harvested at D7, D30 and D90 after the vaccine boost. A Left : Representative zebra plot showing gating strategy after intravenous injection of anti-CD8 α to distinguish between lung parenchyma CD8$^+$T cells (CD8α- CD8β+) and vasculature CD8$^+$ T cells (CD8α+ CD8β+) (Left). A Middle : Frequency of K^b-OVA positive tetramer CD8$^+$T cells gated on live intra-parenchyma CD8$^+$T cells. A right : % of resident memory T cells (TRM) in K^b-OVA positive intra-lung parenchyma CD8$^+$T cells. B : absolute number of intraparenchyma K^b-OVA tetramer positive CD8$^+$T cells at various times after immunization. C : frequency of TRM within K^b-OVA-tetramer positive CD8$^+$T cells gated on intra-parenchyma CD8$^+$T cells. D : absolute number of TRM or effector T cells within K^b-OVA tetramer positive CD8$^+$T cells gated on intra-lung parenchyma CD8$^+$T cells. Mean ± sem with 6 to 9 mice/group were represented in each figure panel. The results are representative of two experiments (n=4/experiment). Values are expressed as means + SEM

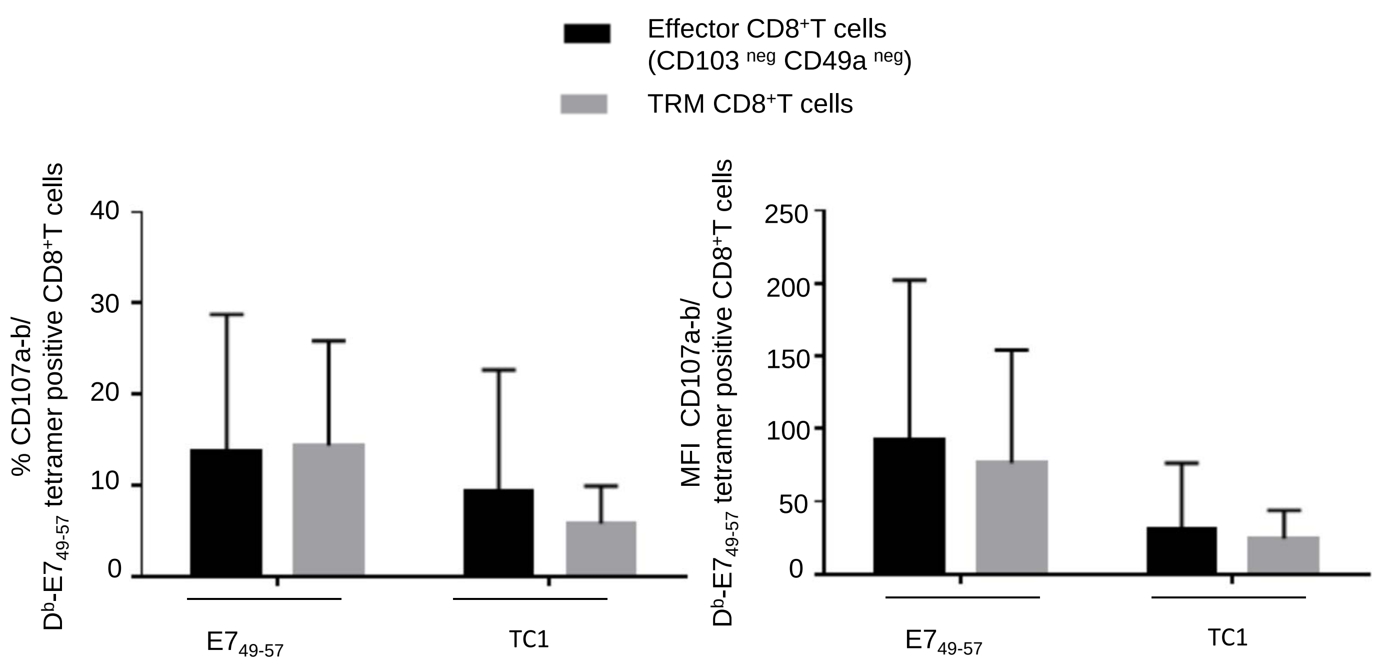

Supplementary Figure 4 : Comparative analysis of CD107 expression after activation of effector $CD8^+$T cells and TRM with specific antigen. Mice (n = 4) were immunized with STxB-E7 at D0 and D14 and cells from LBA were collected at day 21. They were then transferred in wells of round-bottom 96-well plates, and incubated 5h at 37° with the $E7_{49-57}$ peptide (10 µg/ml) or TC-1 cells (10^5 cells/well) in medium containing 1% golgiplug and 1% golgistop and anti- mouse CD107a FITC (0,1ug/well) and anti- mouse CD107b FITC (0.5µg/well). Cells incubated with medium alone served as negative control. After washings, cells were incubated 5 min with Fc receptor block CD16/CD32, then stained with D^b-$E7_{49–57}$ tetramer-PE for 45min at 4° followed by a staining with anti-CD8, anti-CD103, anti CD49a. All the cells were labeled using the live/dead cell viability assay and analysis performed on gated viable cells. Background with medium alone (always < 10%) was subtracted from the results shown. Values are expressed as means + SEM. The results are representative of two experiments (n=4/experiment). .

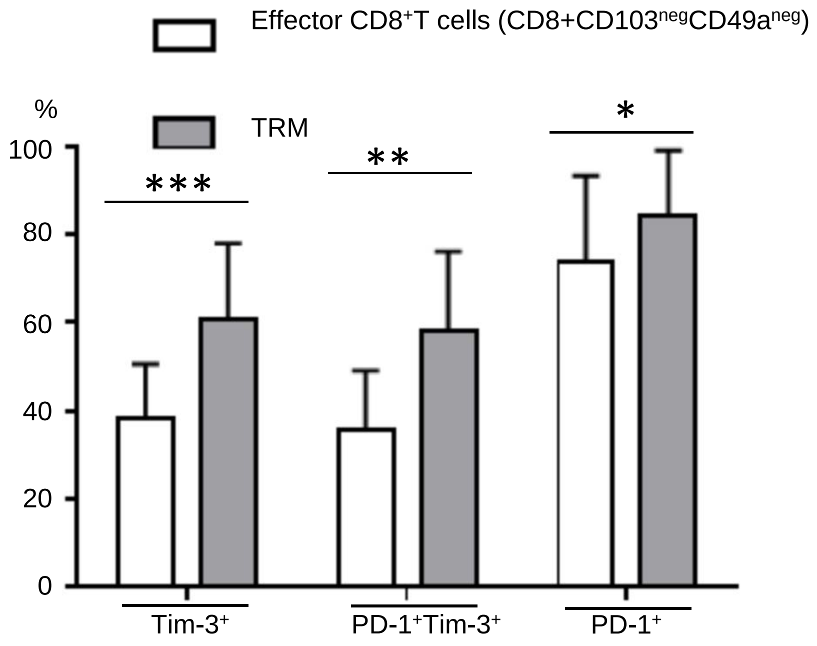

Supplementary Figure 5 : Preferential expression of PD-1 and Tim-3 on intratumoral $E7_{49\text{-}57}$ specific resident memory $CD8^{+}$T cells

Mice were grafted with TC-1 and vaccinated at D5 and D10 with STxB-E7. Tumors were collected at day 14 and after cell dissociation, the cells were stained with live dead, CD45, CD8, D^{b}-$E7_{49–57}$ tetramer , CD103, CD49a, PD-1 and Tim-3. Expression of PD-1 and Tim-3 were compared between effector $CD8^{+}$T cells and TRM ($CD103^{+}$ and/or $CD49a^{+}$). Results shown are representative of two experiments (n=4/experiment). Values are expressed as means + SEM. Paired t-test was selected for statistical analysis . * $p < 0.05$, ** $p < 0.01$, *** $p < 0.001$

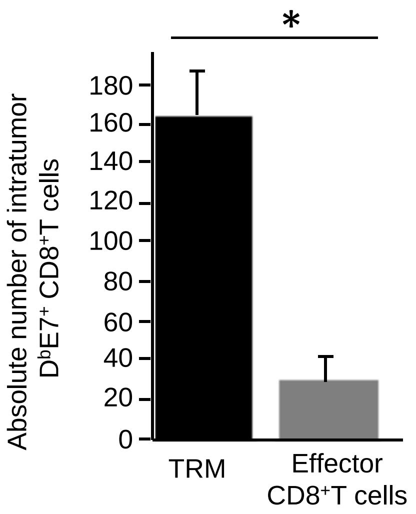

Supplementary Figure 6 : Increased concentration of intratumor TRM over effector CD8^{+}T cells after vaccine administration

Mice were i.n immunized (prime (D0)-boost (D14)) by STxB-E7 and grafted at day 30 with TC-1 in the tongue. At day 35, the number of TRM and effector CD8+T cells were measured in the tongue by flow cytometry. Results shown are representative of three experiments (n=4/experiment). Values are expressed as means + SEM. A non parametric Mann Whitney test was used for statistical analysis * $p < 0.05$

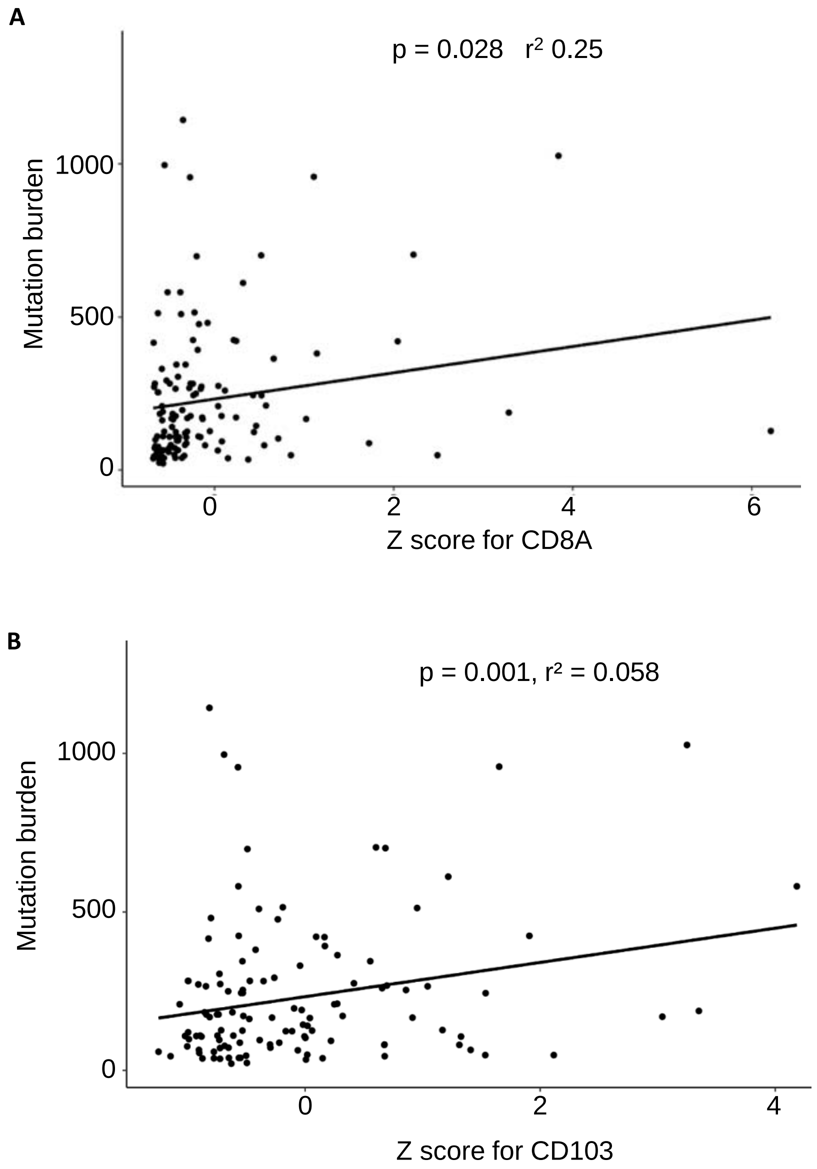

Supplementary Figure 7 : Correlation between CD8A and CD103 gene expression and mutation burden in the TCGA data set for lung adenocarcinoma.

The mutation burden was computed as the distinct number of non-silent mutation for each patient. Expression levels of CD8A (A) and CD103 (B) have been normalized using the z-score transformation. Using a linear regression model, we detect a significant relationship between gene expression and mutation burden

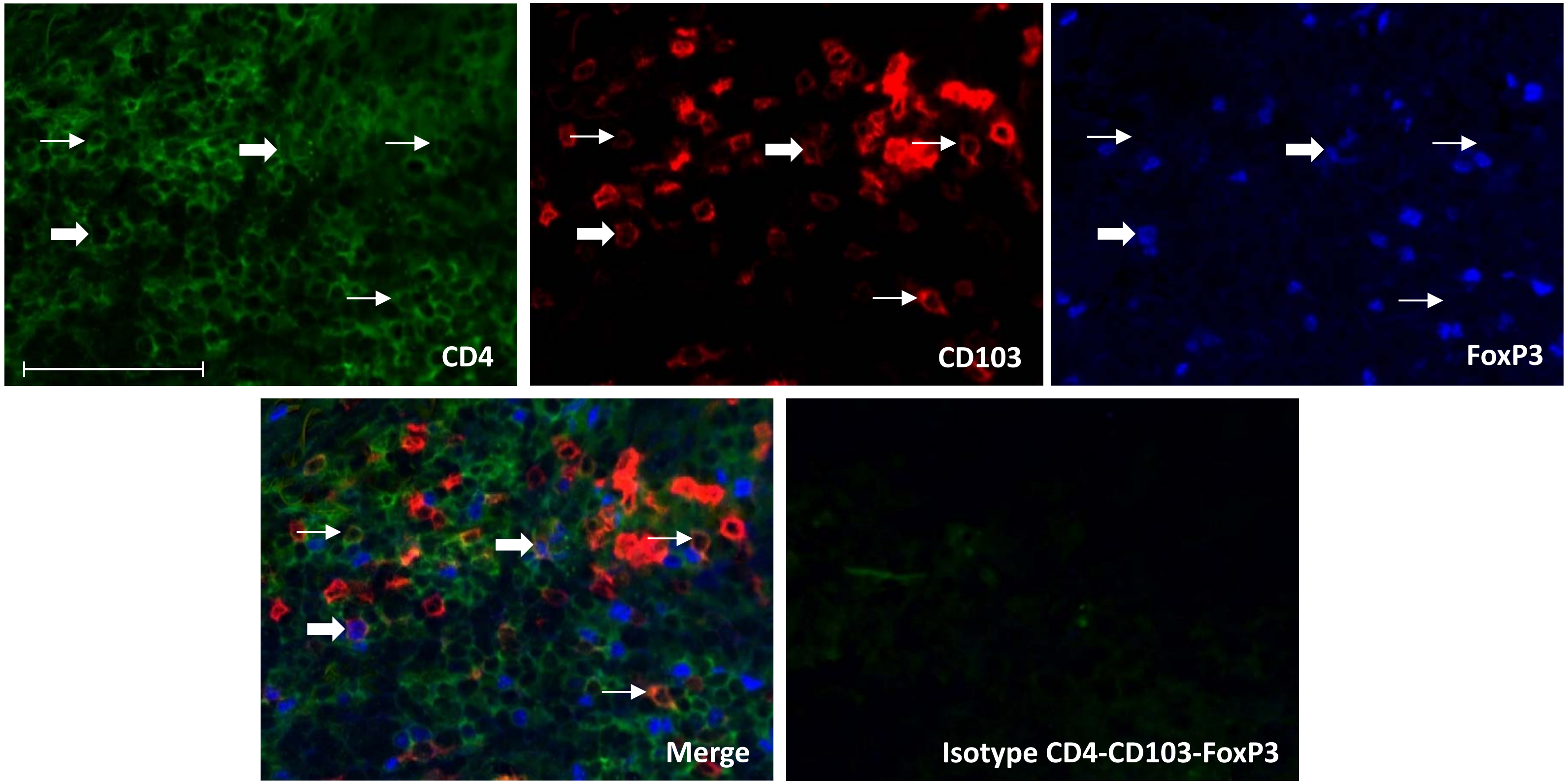

Supplementary Figure 8 : CD103 could be expressed by $CD4^+$T cells and regulatory $FoxP3^+CD4^+$T cells

Frozen tissue section derived from lung carcinoma patients were stained by immunofluorescence with antibodies directed against CD4 (green), CD103 (red) and FoxP3 (blue). The colocalization of CD4, CD103 and FoxP3 markers can be detected by merging the mono-staining picture. The thick arrow indicate co-expression of CD4, CD103 and FoxP3 corresponding to regulatory $CD4^+$T cells and the thin arrow indicate the co-expression of CD4 and CD103 without FoxP3. Staining with isotype controls were included in each experiment (original magnification x 200, scale bar = 100µm)

Characteristics	Number of patients (%)
**Median age (n=96)**	63.7 ±10.0
**SEXE (n=96)**	
Female	25 (26%)
Male	71 (74%)
**Histology (n=96)**	
ADK	60 (62.5%)
SCC	36 (37.5%)
**Stade (n=96)**	
1-2 (localized disease)	45 (46,8%)
3-4 (advanced disease)	51 (53,1 %)
**pTNM (n=96)**	
pT1-2	56 (58,33%)
pT2-3	40 (41,6%)
pN-	44 (46.8)
pN+	50 (53,2%)
pM-	89 (92.7%)
pM+	7 (7.3%)
**Tabacco (n=83)**	
No smoker	14 (16.9%)
Smoker	69 (83.1%)
**Treatment**	
Neo-adjuvant chemotherapy (n=96)	7 (7.3%)
Adjuvant chemotherapy (n=74)	36 (48.6%)
Radiotherapy (n=72)	17 (23.6%)
**Survival**	
Median PFS.in months (n=77)	32.3 [13.8, 47.5]
Median OS.in months (n=96)	36.3 [22.9, 50.1]

Supplementary Table 1 : Patient characteristics

Immunofluorescence analysis in human specimen	Conjugated antibodies	Concentrations
**CD8-CD103-E-cadherin**	FITC conjugated mouse IgG1 κ anti-human CD8a , clone HIT8a , 11-0089 (eBioscience)<br>PE conjugated mouse IgG1 κ anti-human CD103 , clone B-Ly7 , 12-1038 (eBioscience)<br>AF647 conjugated mouse IgG1 κ anti-human E-Cadherin, clone 67A4, 324112 (Biolegend)	10µg/mL<br>5µg/mL<br>5µg/mL
**CD8-CD49a-E-cadherin**	FITC conjugated mouse IgG1 κ anti-human CD8a , clone HIT8a , 11-0089 (eBioscience)<br>PE conjugated mouse IgG1 κ anti-human CD49a, clone TS27, 328304 (Biolegend)<br>AF647 conjugated mouse IgG1 κ anti-human E-Cadherin, clone 67A4, 324112 (Biolegend)	10µg/mL<br>20µg/mL<br>5µg/mL
**CD4**	Unconjugated goat IgG anti-human CD4 (AF-379-NA) RαD<br>Secondary antibody donkey anti-goat FITC Jackson ImmunoResearch (705-095-147)	10µg/mL<br>10µg/mL
**Foxp3**	APC conjugated mouse IgG2a κ anti-human FoxP3 (PCH101, 17-4776) eBioscience	10µg/mL

Supplementary Table 2 : List of antibodies used for in situ immunofluorescence staining

	Lymphocytes ($10^3/\mu l$)	Normal value ($10^3/\mu l$)
Control mice	3.52	0.9-9.3
Mice treated with FTY720	0.09	0.9-9.3

Supplementary Table 3 : Mice treated with FTY720 developed a lymphopenia
Tumor bearing mice were treated with FTY720 and blood was collected 24 hours after its administration. These experiments have been done twice with 5 mice per group. Mean is represented.
